# Cathepsin S: molecular mechanisms in inflammatory and immunological processes

**DOI:** 10.3389/fimmu.2025.1600206

**Published:** 2025-07-07

**Authors:** Huan Gao, Zirui Zhang, Jiayu Deng, Yanqing Song

**Affiliations:** ^1^ College of Pharmacy, Jilin University, Changchun, Changchun, China; ^2^ Department of Clinical Pharmacy, the First Hospital of Jilin University, Changchun, China; ^3^ Department of Pharmacy, Lequn Branch, the First Hospital of Jilin University, Changchun, China

**Keywords:** cathepsin S (CTSS), molecular mechanism, immunoregulation, inflammatory disease, therapeutic targets

## Abstract

Cathepsin S (CTSS), a lysosomal cysteine protease predominantly expressed in immune cells, governs inflammatory and immunological cascades through proteolytic activity. Beyond maintaining lysosomal proteostasis through protein degradation, CTSS executes dual immunomodulatory functions: intracellularly processing antigen-presenting molecules and modulating inflammatory signaling cascades; extracellularly activating protease-activated receptors (PARs) and remodeling the extracellular matrix (ECM). Its dysregulation drives pathology in autoimmune disorders, chronic inflammation, and neoplasia, establishing CTSS as a multifaceted therapeutic target. This review comprehensively explores the contributions of CTSS signaling in immune-mediated inflammatory diseases, critically evaluates its therapeutic potential, highlighting its significance in the development of innovative treatment strategies.

## 1 Introduction

Inflammation represents a critical pathophysiological response to diverse stimuli including infections, tissue injury, cellular stress, and chemical agents. Immune cells orchestrate inflammatory processes through coordinated mechanisms: circulating leukocytes infiltrate damaged tissues, while resident macrophages modulate local inflammatory dynamics (1). Pathological inflammation underlies diseases, ranging from immune-mediated disorders (such as multiple sclerosis and Crohn’s disease) to cardiovascular pathologies (e.g., atherosclerosis), neurodegenerative conditions (e.g., Alzheimer’s disease), and psychiatric disorders (e.g., generalized anxiety disorder) (2–9). Within cellular microenvironments, inflammatory signaling is regulated by lysosome-dependent mechanisms, where macrophages and dendritic cells (DCs) employ lysosomal enzymes as key effectors (10). Among these, cathepsins (CTSs) mediate inflammatory signal transduction through their proteolytic processing of intracellular and extracellular proteins.

Cathepsin S (CTSS), a lysosomal cysteine protease, uniquely maintains enzymatic activity at neutral pH (≤7) due to its histidine-rich propeptide domain, distinguishing it from acid-dependent cathepsins (e.g., Cathepsin B/L/K) (11). This property enables CTSS to perform both intra- and extracellular functions. Intracellularly, lysosomal membrane permeabilization (LMP) releases CTSS into the cytosol, triggering lysosome-dependent cell death (12, 13). Extracellularly, lysosomal exocytosis facilitates CTSS secretion, enabling ECM remodeling, receptor activation (e.g., PAR2), and cytokine release (14, 15). Recent studies highlight CTSS as a central regulator of inflammatory pathways. CTSS is required for Major Histocompatibility Complex class II (MHC-II) maturation in antigen-presenting cells (including DCs), activating adaptive immunity (16–18). In addition, CTSS deficiency disrupts autophagic flux, leading to autophagosome accumulation and pro-inflammatory signaling that amplifies inflammatory responses (18–21). Crucially, extracellular CTSS activates Protease-activated receptor 2 (PAR2) and fractalkine (FKN) —key mediators in autoimmune disease pathogenesis (22, 23). The mechanisms contributing to anti-inflammatory effects are manifold. Despite these multifaceted roles, a systematic discussion of CTSS-mediated signaling networks remains lacking.

In this paper, we summarized upstream regulators of CTSS signaling, downstream inflammatory mediators and associated diseases. We then evaluate CTSS as a therapeutic target to inform novel treatment strategies for inflammatory disorders.

## 2 Stress factors upregulating CTSS expression

**Figure 1:**
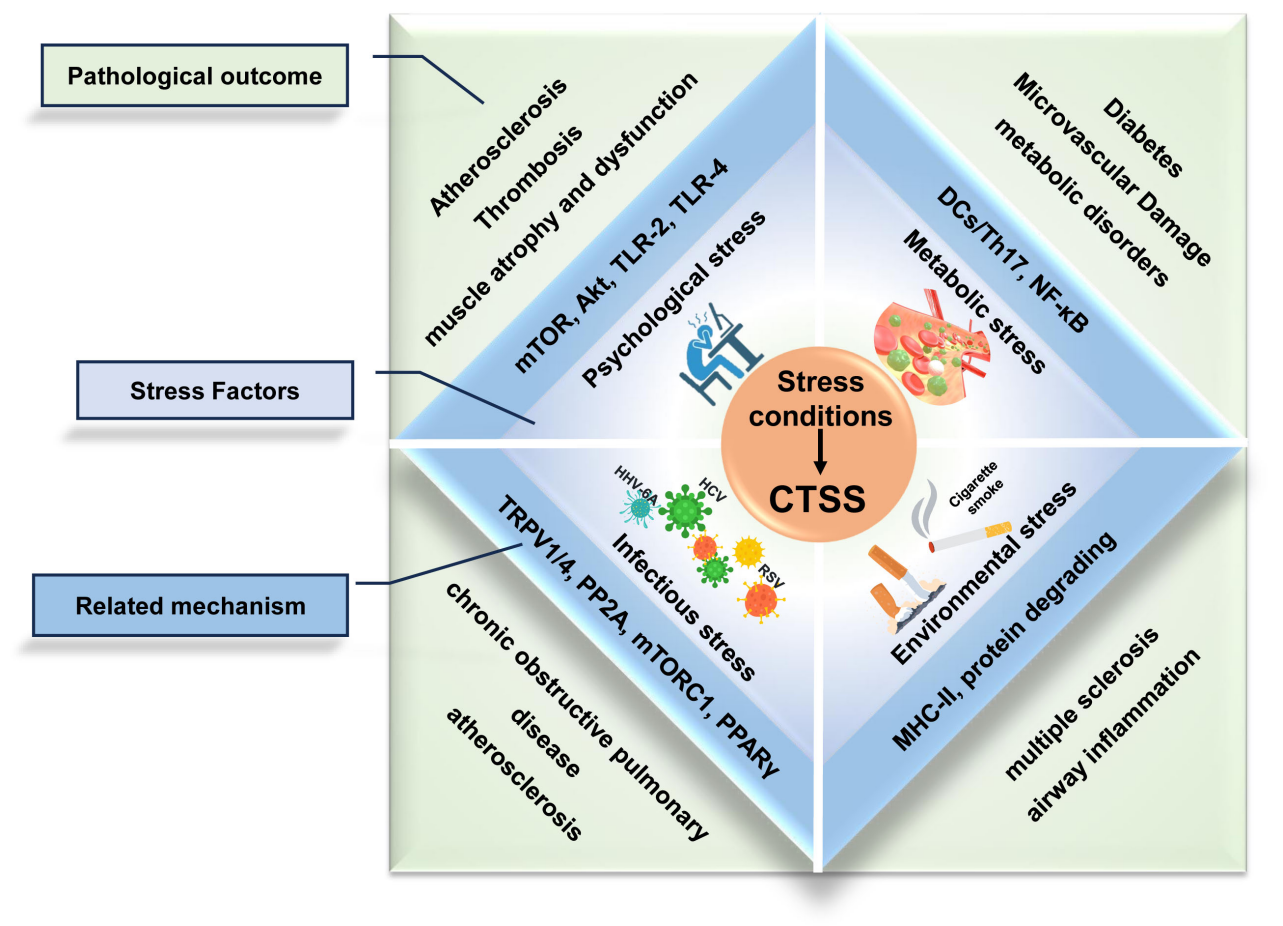
Stress-induced upregulation of CTSS drives inflammatory disease progression.

Stress conditions act as pathological factors which contribute to inflammation micro-environment and promote immunological diseases progression (**Figure 1**). CTSS overexpression induced by psychological, metabolic, or infectious stress correlates with disease progression, reinforcing its therapeutic relevance.

The major stress conditions (psychological, metabolic, environmental and infectious stress) converge to promote pathological overexpression of CTSS. This upregulation establishes a pro-inflammatory microenvironment characterized by dysregulated immune responses, protease activation, and tissue damage.

### 2.1 Psychological Stress

Chronic psychological stress (CPS) refers to persistent physiological dysregulation induced by exogenous stressors (e.g., occupational or social pressures), which can trigger systemic chronic inflammation (24, 25). CTSS upregulation correlates with CPS progression, driving pathologies like atherosclerosis and muscle atrophy. Notably, CTSS promotes upregulation of inflammatory factors (mTOR, Akt, TLR-2, TLR-4, Bcl-2, SOD, Caspase-3, MMP-2, MMP-9, MCP-1, and p-GSK3α/β), enhancing apoptosis, oxidative stress, and macrophage infiltration (26–28). Moreover, depending on the type of inflammation, CTSS can induce unique pathological changes. In stress-aggravated atherosclerosis, it promotes plaque elastin disruption, smooth muscle cell proliferation/migration, and neointimal hyperplasia. Conversely, stress-related thrombosis events involve endothelial loss and the activation of pro-coagulation factors (PAI-1, ADAMTS13, and vWF) (26, 27). For chronic stress-induced muscle atrophy and dysfunction, CTSS contributes to the loss of myotube myosin heavy chain content and the upregulation of Muscle RING-finger protein-1 (MuRF1) and Insulin Receptor Substrate 2 (IRS-2) (28). These distinct mechanisms demonstrate how CTSS upregulation advances CPS-related inflammation through pro-inflammatory effects and tissue-specific actions, establishing its role as a critical therapeutic target.

### 2.2 Metabolic stress

Hyperglycemia is a well-established risk factor for diabetes and its complications, including chronic inflammation and microvascular pathologies (29, 30). In diabetic models, it elevates CTSS levels in DCs within perivascular adipose tissue, which subsequently drive T helper 17 cell (Th17) differentiation and pro-inflammatory cytokine production (e.g., IL-6). Conversely, CTSS knockdown mitigates hyperglycemia-induced carotid restenosis, confirming immune cell-mediated CTSS involvement in microvascular complications (30). Hyperglycemia also directly upregulates CTSS in endothelial cells. CTSS activates nuclear factor kB (NF-κB) signaling and then triggering inflammatory cytokine release (TNF-α, IL-1β, IL-6), angiogenic factor overexpression (MCP-1, VEGFA, VCAM-1), and complement system activation (C3a, C5a) (29). These synergistic effects exacerbate endothelial injury, establishing CTSS as a dual mediator of hyperglycemia-induced vascular damage. Clinically, the association has been proved in a study of investigating CTSS and type 2 diabetes. Each baseline CTSS standard deviation increase correlates with 41- 48% higher diabetes risk across multivariable models (31). CTSS inhibition could alleviate hyperglycemia-induced endothelial inflammation *in vitro*, and reduce hepatic glucose production in murine models, while maintaining unaltered glucose metabolism in skeletal myotubes and adipocytes (29, 32). In summary, CTSS emerges as a critical molecular link between hyperglycemia and complications, positioning it as a promising target for both hyperglycemia prevention and complication management.

### 2.3 Environmental stress

Cigarette smoke contains multiple pro-inflammatory constituents, including nicotine, which induces systemic inflammatory damage across respiratory and cardiovascular systems, thereby promoting pathogenesis of chronic obstructive pulmonary disease (COPD) and atherosclerosis (33). Nicotine activates CTSS-dependent inflammatory pathways through distinct tissue-specific mechanisms. In pulmonary tissues, nicotine triggers Transient Receptor Potential Vanilloid 1/4 (TRPV1/4) receptors on alveolar macrophages, inducing P2X purinoceptor 7 (P2X7)-dependent intracellular calcium influx that stimulates p38/MAPK phosphorylation and enhances lysosomal CTSS production. The CTSS Overexpression amplifies oxidative stress, promotes macrophages recruitment, and disrupts proteases/anti-proteases balance, collectively damaging lung homeostasis to drive COPD onset and progression (34). CTSS-deficient mice demonstrated protection against smoke-induced pathologies including pulmonary inflammation, airway hyperresponsiveness, emphysema, and lung function decline, while Protein Phosphatase 2A (PP2A) activation suppresses CTSS-driven pulmonary disorders (35). Notably, endogenous nitrated fatty acids (NFAs) exhibit therapeutic potential by activating Peroxisome Proliferator-Activated Receptor γ (PPARγ) signaling and Cys25 S-alkylation modification of CTSS, effectively reversing nicotine-induced inflammation (36). In the cardiovascular system, nicotine inhibits the Mechanistic Target of Rapamycin Complex 1 (mTORC1) to promote Mitochondrial Transcription Elongation Factor B (TEFB) nuclear translocation and CTSS transcription. Conversely, nicotine-mediated mTORC1 inhibition also contributes to CTSS secretion by enhancing the Ras-related protein Rab-10-mTORC1 interactions to facilitate lysosomal exocytosis. CTSS overactivity disrupts vascular smooth muscle cell migration, contributing critically to atherosclerotic plaque formation (37). This mechanistic analysis identifies CTSS as a central mediator linking cigarette smoke to multi-organ inflammation. Though pharmacological CTSS inhibition represents a promising therapeutic strategy, smoking cessation remains the most effective preventive measure against CTSS-mediated systemic damage.

### 2.4 Infectious stress

CTSS drives viral-associated inflammatory diseases progression through pathogen-specific proteolytic mechanisms. In neuroinflammatory disorders, human herpesvirus-6A (HHV-6A) induces CTSS release from neural cells, directly degrading myelin basic protein to initiate demyelination cascades characteristic of multiple sclerosis (38). Respiratory syncytial virus (RSV) synergizes with cigarette smoke in COPD pathogenesis by coordinated upregulating CTSS expression, exacerbating airway inflammation via ECM remodeling (39). Similarly, hepatitis C virus (HCV) employs its NS5A core protein to disrupt Interferon-γ (IFN-γ)/(interferon regulatory factor 1 (IRF-1) signaling, inducing CTSS dysregulation that impairs MHC-II-mediated antigen presentation in hepatic DCs (40). These findings establish CTSS as a common pathogenic effector across diverse viral infections, with therapeutic implications for attenuating infection-associated immunopathology. Divergently, bacterial pathogens like *Brucella abortus* employ distinct mechanisms, suppressing MHC-II maturation through lipoprotein-dependent impairment of early antigen-presenting complex formation via CTSS-independent pathways (41). Collectively, CTSS emerges as a mediator of viral-associated inflammation and chronic disease transformation, though its pathogenic role in bacterial infections warrants systematic investigation.

## 3 CTSS mediated inflammatory signaling pathways

### 3.1 CTSS regulation in response to inflammatory transcriptional factors upstream

**Figure 2:**
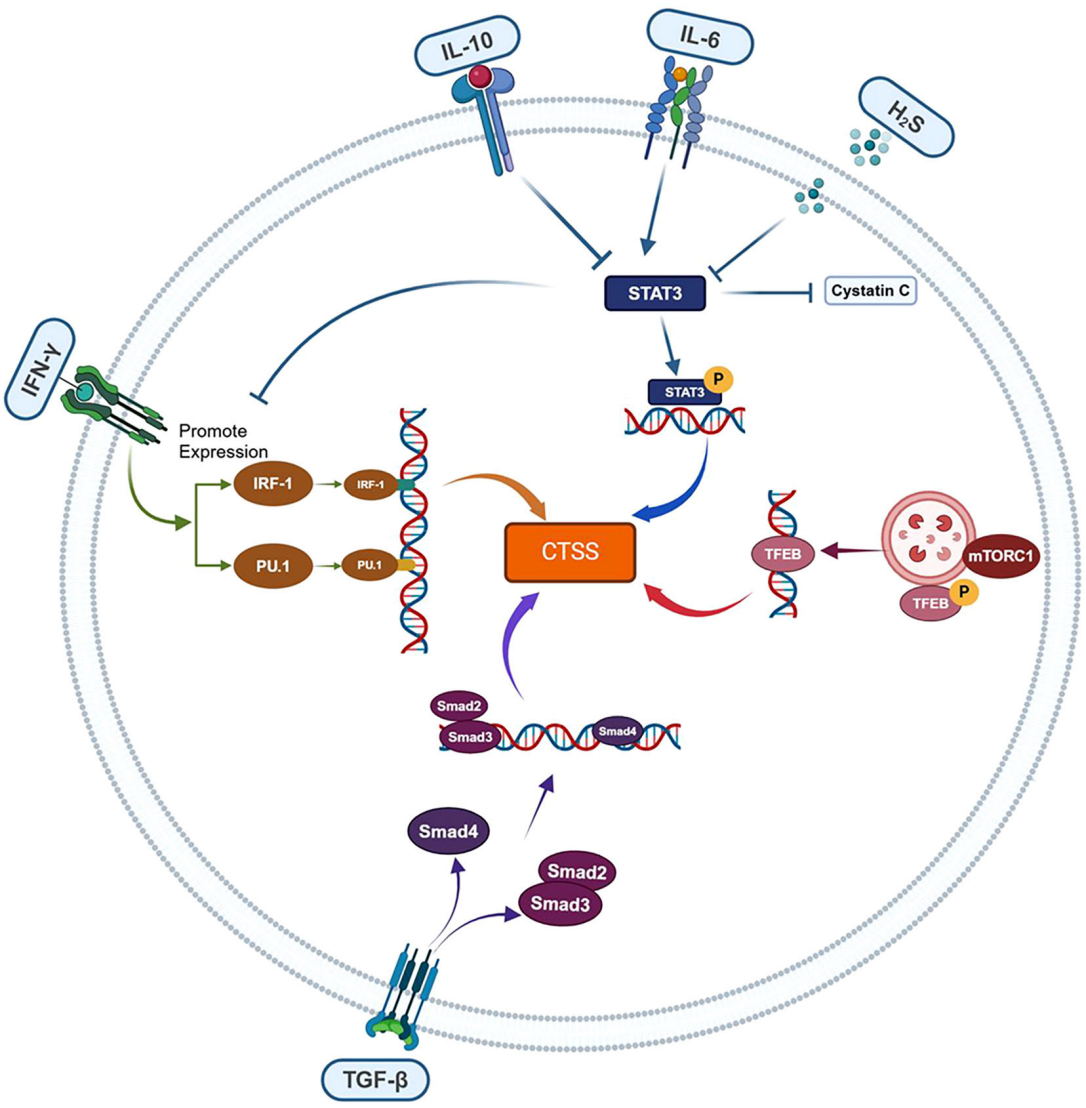
CTSS regulation by upstream inflammatory transcriptional factors. CTSS expression is dynamically modulated by inflammatory transcription factors with opposing regulatory effects: IRF-1, PU.1 and TFEB act as transcriptional activators, whereas TGF-β/Smad function as repressors. STAT3 exerts context-dependent effects, influenced by upstream signals (e.g., IL-6/IL-10 pro-/anti-inflammatory cytokines or H_2_S).

CTSS is a central mediator bridging upstream transcriptional regulators and downstream inflammatory outputs (**Figure 2**). Some transcription factors upregulate CTSS expression (IRF-1, PU.1, TFEB), while others perform as the downregulator (TGF-β/Smad). However, there are also some regulatory signals (Signal Transducer and Activator of Transcription 3, STAT3 etc.) with both the potential effects. The regulation of CTSS by these transcription factors depends on the pro/anti-inflammatory properties of upstream cytokines (IL-6, IL-10 etc.) or other signaling molecules (H_2_S etc.).

#### 3.1.1 IRF-1

IRF-1 is a member of the interferon regulatory factor (IRF) family, critically promotes CTSS transcription (42). Its activity is tightly regulated by upstream cytokines, particularly IFN-γ. The IFN-γ/IRF-1/CTSS axis has been extensively studied in inflammatory pathologies including bronchial inflammation, radiation-induced oxidative stress, and hepatitis C (43). Specifically, IFN-γ upregulates IRF-1 expression, activating CTSS transcription via binding to the interferon-responsive sequence element (IRSE) promoter. Radiation-induced oxidative stress enhances IFN-γ release (44), whereas the HCV core protein N5SA suppresses IFN-γ activity (40), thereby modulating IRF-1/CTSS signaling. Conversely, in *Brucella abortus*-infected monocytes, bacterial lipoproteins downregulate IRF-1 through IL-6 signaling activation, reducing CTSS levels (41). Beyond cytokines, microRNAs have been implicated in the modulation of IRF-1 expression. In cystic fibrosis lung tissues, miR-31 expression is significantly reduced. Treatment with miR-31 analogs suppresses both IRF-1 expression and CTSS secretion, confirming miR-31-mediated negative regulation the IRF-1/CTSS signaling axis (45). Pharmacological agents also modulate this axis. Oxaliplatin upregulates IRF-1 expression in peripheral nerves, activating CTSS/store-operated calcium entry (SOCE) signaling to drive neuroinflammation (46). Thus, the IRF-1/CTSS pathway could be modulated by cytokines, microRNAs, and pharmaceuticals, which mediates inflammatory pathology in radiation injury, infections, genetic disorders, and pharmacogenetic diseases.

#### 3.1.2 PU.1

PU.1, an E26 transformation-specific sequence (ETS) family transcription factor, is essential for the development and differentiation of immune cells, including B cells, macrophages, and neutrophils while disrupting fibrotic networks to promote multi-organ fibrosis regression (47). PU.1 enhances antigen presentation in macrophages, DCs, and B lymphocytes by upregulating CTSS expression, which is critical for MHC-II activity. In the periodontitis model, PU.1-mediated CTSS regulation in macrophages correlates with inflammatory pathway activation (e.g., p38 and NF-κB signaling) and elevated levels of inflammatory factors (e.g., IL-6) (48). PU.1 initiates CTSS transcription through direct binding to ETS motifs in its promoter (49), with all three protein domains driving promoter activity—explaining reduction in CTSS levels observed in PU.1-knockdown macrophages (50). Notably, PU.1 and IRF-1 exhibit co-regulatory behaviors in CTSS expression. Both transcription factors are upregulated by IFN-γ and possess binding sites on the CTSS promoter. Additionally, PU.1 can interact with other transcription factors, including IRF-1, IRF-4, and IRF-8, forming complexes that synergistically enhance CTSS expression (51). Thus, PU.1 drives CTSS expression both directly through promoter binding and cooperatively via transcription factor partnerships. Critically, PU.1-dependent CTSS induction mediates periodontitis progression, underscoring its pathogenic role in inflammatory disease mechanisms.

#### 3.1.3 STAT3

STAT3 is a pivotal regulator of inflammatory pathologies, exerting context-dependent effects on disease progression. Under pro-inflammatory conditions (e.g., IL-6/JAK signaling), STAT3 phosphorylates and dimerizes (p-STAT3), translocating to the nucleus to upregulate CTSS expression while suppressing cystatin C transcription. This dual regulation amplifies CTSS activity, as demonstrated in DCs (52). Conversely, anti-inflammatory signals differentially modulate the STAT3/CTSS axis: IL-10 enhances STAT3 activation via a non-canonical pathway but inhibits IFN-γ-driven CTSS upregulation in macrophages. While both IL-6 and IL-10 upregulate STAT3 activity, they exert different effects on CTSS expression through distinct pathways (53). Notably, endogenous hydrogen sulfide (H_2_S) suppresses this axis through dual mechanisms (1): Cys-259 persulfidation of STAT3 impairs phosphorylation-dependent activation, indirectly reducing CTSS expression (2); Direct Cys-25 persulfidation of CTSS attenuates its enzymatic activity (54, 55). These regulatory networks are functionally validated in models spanning arterial calcification, elastin degradation, and neuroinflammation (54–56). In addition, the STAT3/CTSS signaling axis is implicated in diseases such as Alzheimer’s disease, diabetic nephropathy, and vascular calcification, highlighting its therapeutic potential in cardiovascular and neurological disorders (57–59).

#### 3.1.4 Others

Lysosomes critically regulate CTSS activity. Nicotine impairs mTORC1-mediated autophagy-lysosomal pathway, triggering TFEB nuclear translocation that elevates CTSS transcription and promotes chronic inflammation in atherosclerosis (37). In lupus pathology, Blimp-1 suppresses CTSS expression and inhibits CTSS-mediated MHC-II activation in DCs, blocking antigen presentation to CD4+ follicular helper T cells (60). The TGF-β/Smad pathway exhibits different regulation of CTSS: Chen et al. demonstrated that TGF-β1/Smad2/Smad3/signaling promotes cardiac fibroblasts dedifferentiation via CTSS, upregulating ECM-associated proteins to exacerbate post-infarction fibrosis (61). Conversely, Zhang et al. found that TGF-β/Smad4 inhibition increases CTSS-dependent ECM remodeling, conferring protection against aortic aneurysms (62). This dual functionality highlights the TGF-β/Smad/CTSS axis complexity, warranting further mechanistic investigation.

### 3.2 CTSS passing inflammatory signal to targets downstream

**Figure 3:**
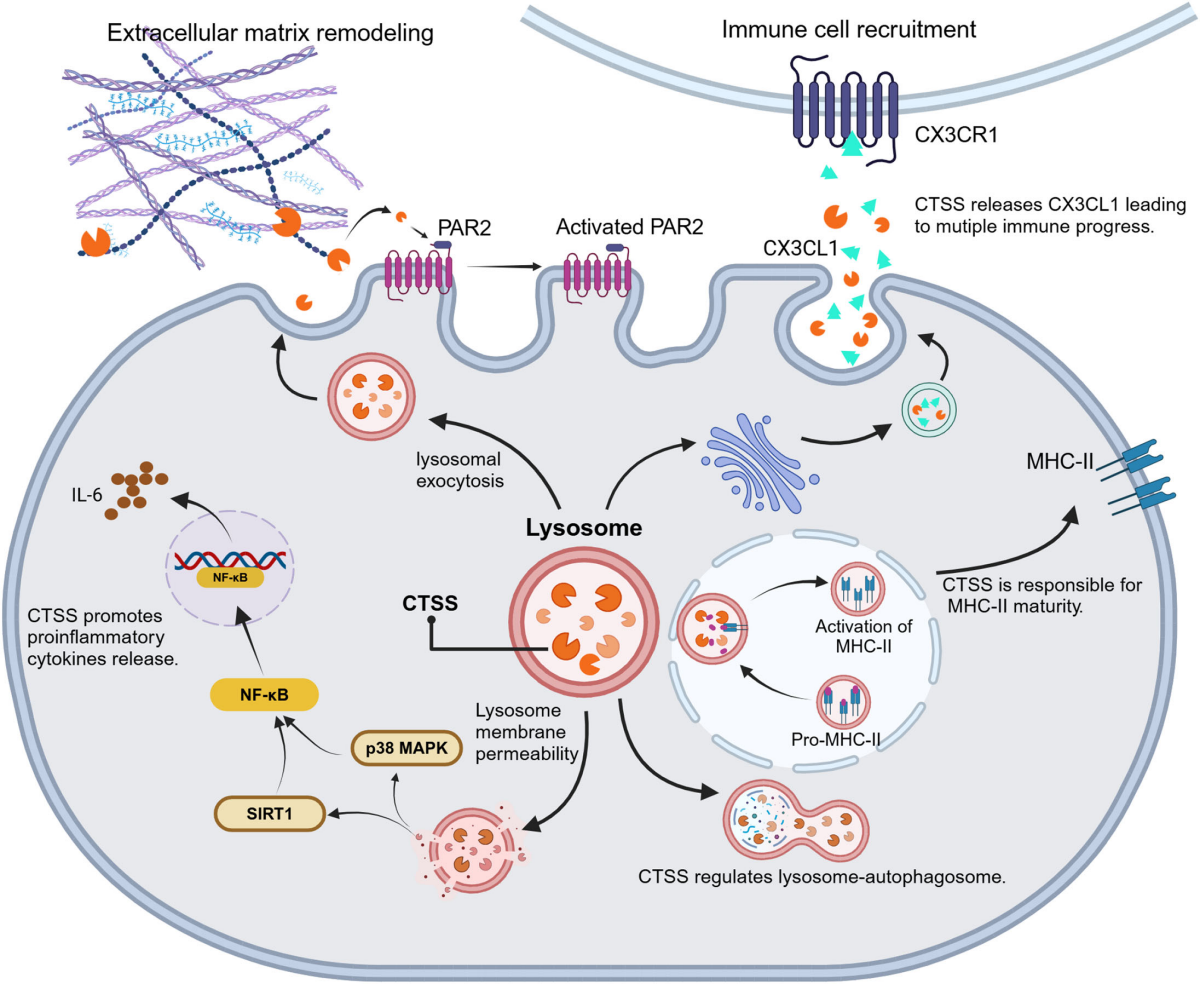
CTSS-driven inflammatory signal transduction. CTSS acts as a proteolytic hub to amplifies inflammatory responses through modulation of transcriptional activators (e.g., NF-κB activation), exposure of cryptic signaling domains (e.g., PAR2, MHC-II) and release of bioactive inflammatory factors (e.g., FKN/CX3CL1).

CTSS exerts its hydrolysis ability to regulate the levels of proinflammatory transcription (NF-κB etc.), to expose the active domain or site of downstream signal proteins (PAR2, MHC-II etc.) or to liberate inflammatory factors (FKN etc.) as shown in **Figure 3**. Therefore, CTSS performs as an amplifier to transduce inflammatory signals.

#### 3.2.1 PAR2

PAR2, a G-protein-coupled receptor, drives inflammation progression, immune responses, and angiogenesis. CTSS activates PAR2 via cleavage at a specific site (E56-T57), distinct from the typical trypsin cleavage site (R36-S37) (63). This unique proteolysis generates signaling responses divergent from classical PAR2 pathways—calcium mobilization, ERK1/2 activation, β-arrestin recruitment, and endocytosis (64). The CTSS/PAR2 axis exacerbates inflammatory diseases via distinct molecular mechanisms. In colitis models, CTSS/PAR2 signaling activates the damage sensor transient receptor potential vanilloid 4 (TRPV4), resulting in visceral hypersensitivity (65). During atopic dermatitis, CTSS overexpression in DCs upregulates PAR2, enhancing secretion of inflammatory cytokines (IL-2, IL-4, IL-10, IFN-γ, and MCP-1) by Th1 cells and triggering scratching behavior (66). In desiccated ocular surfaces and cystic fibrosis lungs, CTSS/PAR2 signaling elevates pro-inflammatory mediators (IL-6, IL-8, TNF-α, IL-1β, and MMP-9) and mucins, respectively (67, 68). These findings collectively demonstrate that CTSS/PAR2 signaling amplifies inflammatory phenotypes through cytokine hypersecretion, receptor overexpression, and pathological protein accumulation.

#### 3.2.2 FKN

FKN (CX3CL1), a transmembrane chemokine expressed in monocytes, NK cells, T cells, and vascular smooth muscle cells, undergoes CTSS-mediated proteolytic cleavage to generate soluble monomer (sFKN). The sFKN activates the G protein-coupled receptor CX3CR1, regulating immune homeostasis (69, 70). In rheumatoid arthritis models (collagen-induced arthritis, temporomandibular arthritis, and desiccation syndrome), CTSS is essential for sustaining inflammatory pain (23, 69, 71, 72). Mechanistically, peripheral nerve injury triggers microglial CTSS secretion. CTSS hydrolyzes membrane-anchored FKN from dorsal horn neurons, releasing sFKN that activates microglial CX3CR1 to induce p38/MAPK phosphorylation. This cascade upregulates nociceptive mediators (TRPV1, P2X4, BDNF, α2δ calcium channels, and activating transcription factor 3), amplifying pain signaling (57, 73, 74). Notably, P2X7 receptor-dependent CTSS upregulation establishes the P2X7/CTSS/FKN axis as a therapeutic target for inflammatory pain (75). In desiccation syndrome, CTSS/FKN signaling drives dacryoadenitis by recruiting T cells and macrophages into lacrimal gland—blocked by CTSS or FKN inhibition (76). Similarly, neuronal CTSS accelerates Alzheimer’s pathogenesis in murine models by driving microglial M1 polarization, activating the FKN-CX3CR1 axis, and enhancing JAK2-STAT3 signaling. Elevated CTSS in human Alzheimer brains and the therapeutic efficacy of CTSS inhibitor LY3000328 confirm its clinical relevance (57). In summary, the CTSS/FKN axis sustains chronic pain in arthritis and drives autoimmune inflammation through immune cell recruitment across multiple pathologies.

#### 3.2.3 MHC-II

MHC-II complexes on antigen-presenting cells (APCs) enable immune recognition by presenting processed antigens to CD4+ T cells (77, 78). CTSS, the primary invariant chain (li)-processing enzyme, is highly expressed in MHC-II-positive APCs, including DCs, monocytes, lymphocytes, and splenocytes (79). This protease selectively cleaves the li chain within the MHC-II precursor complex (αβli trimer), generating mature αβ-CLIP complex that load exogenous antigens for CD4+ T cell activation and humoral immunity. Beyond antigen presentation, CTSS-mediated li degradation enhances DC motility by disrupting interactions with myosin II, thereby increasing migration speed and directional persistence for efficient antigen detection (18). Targeting CTSS has emerged as a therapeutic strategy for autoimmune diseases (e.g., rheumatoid arthritis, asthma, desiccation syndrome, multiple sclerosis) due to its dual role in MHC-II maturation and DC motility (80–83). Pharmacological inhibition like Clik60 block li degradation, causing accumulation of MHC-II-li intermediates and impairing MHC-II-peptide complex formation in APCs. This compromises antigen presentation while suppressing lymphocyte and eosinophil infiltration across multiple tissues. Consequently, CTSS inhibition alleviates autoimmune pathology through reduced autoantibody production and resolved tissue inflammation.

#### 3.2.4 NF-κB

NF-κB, a master transcriptional regulator of inflammation and immunity, exhibits complex regulation with CTSS. Emerging evidence demonstrates CTSS promotes NF-κB activation in autoimmune encephalomyelitis, hepatitis, periodontitis, and hyperglycemia-induced endothelial inflammation (19, 29, 84). Notably, CTSS and MHC-II collaboratively regulate NF-κB activity—combination of both synergistically suppresses NF-κB-driven inflammation in encephalomyelitis (84). CTSS indirectly modulates NF-κB through distinct pathways (1): Degrading silent information regulator 1 (SIRT1) (an NF-κB suppressor), where CTSS inhibition stabilizes SIRT1 to repress NF-κB in hepatitis (19) (2); Activating PU.1/p38-NF-κB signaling in macrophages to drive IL-6-mediated periodontitis (48). Paradoxically, CTSS deficiency exacerbates angiotensin II-induced cardiac fibrosis by impairing lysosomal degradation. This disruption causes mitochondrial reactive oxygen species accumulation and subsequent NF-κB hyperactivation (85). Collectively, CTSS either amplifies or suppresses NF-κB signaling depending on tissue context and disease state.

#### 3.2.5 Other

Recent studies indicate CTSS regulates additional inflammatory signaling pathways beyond established mechanisms, though some findings require further validation. In oxaliplatin-induced neuroinflammation, CTSS inhibition activates Stromal Interaction Molecule 1-mediated SOCE, which upregulates anti-inflammatory IL-10 through IRF-1 signaling to alleviate neuropathic pain (46). CTSS also contributes to hepatic fibrosis via ECM remodeling: Kupffer cell-derived CTSS cleaves collagen 18A1, liberating endothelial inhibitory peptides that activate integrin α5β1 on hepatic stellate cells and accelerate fibrogenesis (86). These findings position CTSS as a promising therapeutic target for both neuroinflammatory disorders and fibrotic diseases.

### 3.3 Clinical application of CTSS inhibitors

Small-molecule CTSS inhibitors have progressed to clinical trials across autoimmune and inflammatory disorders, demonstrating diverse therapeutic potential, as shown in **Table 1**. Petesicatib (RO5459072; Hoffmann-La Roche), a covalent reversible inhibitor, achieved phase II efficacy in Sjögren’s syndrome and phase I evaluation for celiac disease via a gluten challenge trial (NCT02679014), with ongoing exploration for idiopathic pulmonary fibrosis (IPF) (87). Another Roche compound, RO5461111, a competitive selective CTSS inhibitor, shows promise in systemic lupus erythematosus but awaits clinical entry (88, 89). Virobay’s oral inhibitors VBY-036 (phase I, neuropathic pain) and VBY-891 (phase II, psoriasis) exhibit tissue-specific targeting, with the latter favoring skin CTSS modulation, alongside preclinical VBY-825 for neuropathic pain and Alzheimer’s disease (90). Eli Lilly’s non-covalent inhibitor LY3000328 reduced plasma CTSS activity in phase I and advanced to phase II for aortic aneurysm; its derivatives also demonstrate immunomodulatory effects in bladder cancer by regulating T-cell activity (91). RWJ-445380 (Johnson & Johnson/Alza) achieved Phase II efficacy in rheumatoid arthritis (with methotrexate) and plaque psoriasis, though structural details remain undisclosed. Sanofi’s SAR114137 faced limitations due to cathepsin K cross-reactivity during phase I osteoarthritis pain trials. Celera’s CRA-028129 completed phase I for psoriasis but lacked further development (92). Preclinical candidates like AM-3840 (Amura; neuropathic pain/RA), CZ-007 (Merck; Chagas disease), and MIV-247 (Medivir; autoimmune/neuropathic pain) further expand the pipeline, alongside novel scaffolds in early discovery (90, 93). These efforts underscore the expanding applications of CTSS inhibition, from autoimmune diseases (Sjögren’s syndrome) to oncology and vascular remodeling, yet highlight critical challenges in optimizing selectivity and minimizing off-target effects for future candidates.

**Table 1:** A list of CTSS inhibitors at different stages of clinical trials.

S/N	Drug Name	Company	Indication	Phase	Clinical Trial Identifier	Reference
1	Petesicatib (RO5459072)	Hoffmann-La Roche	Sjogren’s syndrome	phase II	NCT02701985	Study Details | A Study to Assess the Efficacy of RO5459072 in Participants With Primary Sjogren’s Syndrome | ClinicalTrials.gov, (n.d.) https://clinicaltrials.gov/study/NCT02701985 (94)
			celiac disease	phase I	NCT02679014	Study Details | A Study to Investigate the Pharmacokinetics, Pharmacodynamic Effects, Safety and Tolerability of Repeated Dosing of RO5459072 in Volunteers With Celiac Disease | ClinicalTrials.gov, (n.d.) https://clinicaltrials.gov/studyNCT02679014
2	RO5459072	Hoffmann-La Roche	Celiac Disease	Phase I	NCT02679014	Study Details | A Study to Investigate the Pharmacokinetics, Pharmacodynamic Effects, Safety and Tolerability of Repeated Dosing of RO5459072 in Volunteers With Celiac Disease | ClinicalTrials.gov, (n.d.) https://clinicaltrials.gov/study/NCT02679014
3	VBY-036	Virobay Inc.	Nerve pain	Phase I	NCT01911637	Study Details | Safety Study of VBY-036 in Healthy Volunteers After 7 Days of Oral Dosing | ClinicalTrials.gov, (n.d.) https://clinicaltrials.gov/study/NCT01892891?term=NCT01892891&rank=1
4	VBY-891	Virobay Inc.	Psoriasis (inflammatory autoimmune disease)	Phase II	NCT01947738	Study Details | Safety Study of VBY-891 in Healthy Volunteers After Single or Multiple (7 Days) of Oral Dosing (VBY891P1) | ClinicalTrials.gov, (n.d.) https://clinicaltrials.gov/study/NCT01947738
5	LY3000328.	Eli Lilly	aortic aneurysm	Phase I	NCT01515358	(95, 96)
6	RWJ-445380	Johnson & Johnson	Rheumatoid Arthritis; psoriasis	Phase II	NCT00425321	Safety and Effectiveness Study of RWJ-445380 Cathepsin-S Inhibitor in Patients With Active Rheumatoid Arthritis Despite Methotrexate Therapy, (n.d.) https://clinicaltrials.gov/study/NCT00425321?tab=history&a=12 Study to Investigate the Safety, Tolerability, Absorption, Distribution, Metabolism, and Elimination of RWJ-445380 Administered to Patients With Plaque Psoriasis, (n.d.) https://clinicaltrials.gov/study/NCT00396422?tab=history&a=5
7	SAR-114137	Sanofi	Neuropathic pain, Pain	Phase I		(92)
8	CRA-028129	Celera	psoriasis	phase I		(92)

(Adopted from https://clinicaltrials.gov) (Accessed on 29 May 2025).

## 4 Discussion

CTSS, a lysosomal cysteine protease with restricted immune cell expression, has evolved from a canonical proteinase to a master regulator of inflammatory and immune homeostasis. Unlike ubiquitously expressed cathepsins (B, C, F, H, L, O, X), CTSS exhibits restricted localization, primarily in antigen-presenting cells (B cells, macrophages, DCs) where it drives MHC-II-mediated immune responses through antigen processing. It also operates in epithelial, smooth muscle, endothelial cells, and neutrophils, enabling both intracellular proteolysis and extracellular modulation of inflammatory pathways. This unique tissue specificity and dual functionality underscore CTSS’s irreplaceable role in bridging lysosomal activity with systemic immune regulation. Its unique capacity to operate across pH gradients enables dual-compartment functionality: intracellularly, it processes antigen-presenting molecules and activates NF-κB/MAPK signaling cascades; extracellularly, it cleaves protease-activated receptors and remodels extracellular matrices. CTSS activity is regulated by microenvironment-responsive transcription factors (STAT3), distinguishing it from other cathepsins. It coordinates immune responses through the interconnected pathways: MHC-II antigen presentation, vesicular trafficking-dependent cytokine release, receptor proteolysis-driven signal amplification, and chemokine-mediated leukocyte recruitment. Additionally, it exhibits novel regulatory mechanisms, including pH-dependent enzymatic state switching, redox-sensitive zymogen activation, and miRNA-mediated expression tuning during immune differentiation. These pathways link CTSS to diverse clinical conditions spanning chronic inflammation, autoimmunity, and immunodeficiencies. Clinically, serum CTSS levels serve as biomarkers for interstitial lung disease and uveitis, while preclinical studies validate its therapeutic targeting in multi-organ pathologies affecting pulmonary, hepatic, cardiovascular, and neural systems.

While foundational insights into CTSS-disease associations have been established (97), there remain critical gaps in systematically mapping its molecular mechanisms across immune-inflammatory pathways. This review fills this gap by elucidating the CTSS-mediated signaling networks and highlighting CTSS as a central hub for therapeutic targeting in immune-related pathologies. The current clinical development of CTSS inhibitors shows promise in attenuating inflammatory lesions while preserving homeostatic functions, although further refinement is needed to optimize therapeutic efficacy. By positioning CTSS as a metabolic-inflammatory-immune signaling nexus, this review advances its characterization as a unique therapeutic node capable of coordinated multi-organ modulation, representing a paradigm shift from conventional protease-targeted approaches. Additionally, we propose a therapeutic strategy targeting CTSS through isoform-specific inhibition, subcellular localization control, and activity-state modulation, offering novel precision interventions for inflammation-related pathologies.
